# The emerging role of the microbiome in bladder cancer: prognostic implications and treatment response

**DOI:** 10.1590/1414-431X2026e15526

**Published:** 2026-04-27

**Authors:** J. Côrtes, J.C.S. Trindade, S.R. Rogatto

**Affiliations:** 1 Hospital das Clínicas, Faculdade de Medicina de Botucatu, Universidade Estadual Paulista, Botucatu, SP, Brasil; 2 Programa de Pós-graduação em Ciências Biológicas, (Genética), Instituto de Biociências, Universidade Estadual Paulista, Botucatu, SP, Brasil; 3 Department of Clinical Genetics, University Hospital of Southern Denmark, Vejle, Denmark; 4 Faculty of Health Sciences, University of Southern Denmark, Odense, Denmark

**Keywords:** Bladder cancer, Urinary microbiome, BCG, Probiotics, Metagenome, Epithelial-mesenchymal transition

## Abstract

Bladder cancer (BCa) is a histologically and molecularly heterogeneous disease and is one of the leading causes of cancer death globally. The main risk factors are sex (with incidence 3 to 4 times higher in men), tobacco usage, occupational exposure to carcinogens, and persistent infections, such as those caused by *Schistosoma haematobium*. Urine and the bladder were recently confirmed to be non-sterile, prompting investigations into the urinary and intratumoral microbiomes and their roles in tumor stage, prognosis, and therapy response. In this context, the role of the urinary and intratumoral microbiome in bladder carcinoma is among the most promising areas in translational uro-oncology. Recent evidence demonstrates the presence and diversity of microbial communities in both urine and bladder cancer tissue, with patterns associated with tumor stage and prognosis. Chronic inflammation, genotoxin production, altered carcinogen metabolism, and modulation of the immune microenvironment are biological processes that provide a rationale for the functional role of these microorganisms in the bladder. Furthermore, microbial profiles have been correlated with responses to intravesical therapies (such as BCG - Bacillus Calmette-Guérin) and, potentially, with systemic immunotherapies. The microbiome can help identify predictors of treatment response and potential adjuvant interventions, and offers a non-invasive, translational pathway for diagnosis and surveillance. This review summarizes current evidence on the microbiome in bladder cancer patients and its prognostic and therapeutic potential.

## Introduction

Bladder cancer (BCa) is a complex entity, characterized by molecular, morphological, and histopathological patterns that exhibit pronounced heterogeneity among its subtypes. Globally, it ranks as the 9th most common cancer, with over 613,000 new cases and approximately 220,000 deaths reported in 2022 (1). The National Cancer Institute (INCA - Brazil) ranks bladder cancer 12th in incidence, with an estimated 11,370 new cases between 2023 and 2025 (https://www.gov.br/inca/pt-br). The global distribution of the disease is unequal, being most frequently diagnosed in Southern, Northern, and Western Europe (with the highest incidence in males from Spain and females from the Netherlands), North America, Eastern Europe, and North Africa (1).

Bladder cancer is primarily associated with aging, with most cases diagnosed at age ≥55 years. The disease is rare in younger individuals (under 40 years of age), who generally have a more favorable prognosis (2). BCa also shows sex-based patterns of incidence, affecting men three to four times more frequently than women, with 471,072 new male cases and 142,719 female cases reported, as estimated by GLOBOCAN 2022 (1). In contrast to the incidence, the prognosis of bladder cancer is significantly worse for female patients. Women tend to be diagnosed with high-grade and advanced-stage tumors, resulting in poorer survival and higher risks of progression and recurrence (3). Differences in biological factors, such as anatomy, genetics, epigenetics, sex hormones, immune system, and microbiome composition, have been investigated to clarify sex differences in incidence, prognosis, and therapeutic response (4).

Tobacco smoking accounts for nearly half of BCa cases, with 50-65% occurring in males (5). Numerous tobacco-derived metabolites are excreted in the urine, exposing the bladder epithelium to carcinogens such as 4-aminobiphenyl, 2-naphthylamine, and polycyclic aromatic hydrocarbons, which can induce DNA damage, oxidative stress, and alterations in gene expression (6,7). Occupational exposure to carcinogens, including ortho-toluidine, arsenic, inorganic arsenic compounds, X-ray and gamma-radiation, 2-naphthylamine, 4-aminobiphenyl, and benzidine, is associated with nearly 10% of bladder cancer cases, affecting workers in the rubber, dye, paint, and petroleum industries (8). Additional risk factors include the use of electronic cigarettes, *Schistosoma haematobium* infection, and dysregulation of drug-metabolizing enzymes, such as CYP1B1, GSTM1, and GSTP1 (9).

Approximately 90% of BCa originate from epithelial cells, with urothelial carcinoma of the bladder (UCB) as the predominant subtype (10). UCB displays histological diversity in up to 40% of cases, including variants such as nested, micropapillary, plasmacytoid, sarcomatoid, and others. These variants are often associated with worse clinical outcomes, characterized by locally advanced disease, higher metastatic risk, and reduced therapeutic response (11). Rare histological variants include squamous cell carcinoma, adenocarcinoma, and small cell carcinoma (12).

At diagnosis, bladder cancer is classified as non-muscle-invasive bladder cancer (NMIBC) or muscle-invasive bladder cancer (MIBC), based on detrusor muscle invasion. BCa can also be classified as papillary, solid, or mixed tumors based on the observed morphology (13). NMIBC accounts for ∼75% of UCB and comprises tumors confined to the mucosa or invading the lamina propria (14). NMIBC tumors generally have a favorable prognosis, with survival rates of 70-85% at 10 years for high-grade disease (15). Low-grade NMIBC Ta tumors have an estimated 55% risk of recurrence and a 6% risk of progression to MIBC. In contrast, high-grade T1 tumors have around 45% and 17% risk of recurrence and tumor progression, respectively (15). MIBC is more aggressive, diagnosed in 25% of cases, with a 5-year survival of around 60% (16,17). Metastatic disease can affect the bones, lungs, brain, liver, and lymph nodes, contributing to the disease's higher lethality, with survival reduced to approximately 15 months (18).

At the molecular level, NMIBC is characterized by mutations that activate the RAS-MAPK and PI3K signaling pathways, as well as loss-of-function in chromatin remodeling genes (19). *FGFR3* and *RAS* mutations account for approximately 70% of NMIBC cases, although not concurrently (18). *PIK3CA* is mutated in about 30% of cases, contributing to activation of the PI3K and RAS-MAPK pathways in association with *FGFR3* or *RAS* (18). Chromatin regulators are mutated in more than 65% of NMIBC cases, with emphasis on *KDM6A* and *ARID1A*, which are frequently found in Ta and T1 tumors, respectively (18). MIBC is a tumor rich in mutations associated with loss of cell cycle control and DNA damage repair pathways, which inactivate the *TP53*, *RB1*, *ATM*, and *ECC2* genes or interfere with their respective regulatory genes, such as *MDM2* and *E2F3* amplification (20). In MIBC, mutations that activate the RAS-MAPK and PI3K pathways occur in approximately 70% of cases (18). Interestingly, *FGFR3* mutations are less frequent, but increased expression and fusion to other genes, such as in *FGFR3*-*TACC3,* are commonly detected (20).

Urine is a valuable noninvasive sample for bladder cancer research. In the last decade, advances in Next-Generation Sequencing have overturned the long-held assumption that urine is sterile, revealing the presence of diverse microbial taxa significantly correlated with carcinogenesis, including induction of the inflammatory process, alterations in gene expression, epithelial-mesenchymal transition, generation of reactive oxygen species (free radicals), and modulation of cellular signaling (21-23). This review addresses the current knowledge of the urinary and intratumoral microbiomes in the development, progression, recurrence, and therapeutic response of urothelial carcinoma of the bladder, highlighting the potential of microbiota modulators for disease monitoring and treatment.

## Composition of the normal urinary and bladder cancer microbiome

The bladder and urine of healthy individuals are colonized by diverse bacterial taxa, with well-established differences between the female and male genitourinary microbiotas. The healthy female microbiota is enriched by the phyla Actinobacteria and Bacteroidetes (24), with a predominance of *Lactobacillus* and *Gardnerella* (25), whereas the male microbiota is dominated by *Corynebacterium*, *Staphylococcus,* and *Streptococcus* (26). The phylum Firmicutes is abundant in both sexes (24). Curiously, the microbiological distinction between the sexes may be associated with the discrepancies in bladder cancer incidence (27).

The literature shows inconsistencies in the reported composition of the urinary microbiome in bladder cancer patients. Bučević Popović and colleagues (28) found that these patients have a distinct urinary microbiome, with increased levels of *Fusobacterium*, *Actinobaculum, Facklamia*, and *Campylobacter*. Differences in bacterial taxa among BCa patients may be attributable to selection bias, inclusion of patients of both sexes, broad age ranges, and the lack of distinction between NMIBC and MIBC samples (29,30). Several studies confirmed differences in microbiome richness and diversity between NMIBC and MIBC, with MIBC generally exhibiting reduced taxonomic richness. However, there is no consensus regarding the microbial richness of normal versus tumor bladder samples (31-34). For instance, Wu et al. (33) and Zhang et al. (34) observed higher bacterial abundance in the BCa-associated microbiome than in control samples, whereas Chipollini et al. (31) and Liu et al. (32) reported lower bacterial diversity in the BCa-associated microbiome than in control samples.

Recent studies of the bladder cancer microbiome have shown significant differences between patients and healthy controls, between NMIBC and MIBC subtypes, and between urine and tissue samples, as summarized in Table 1 (30,31,35-38).

**Table 1:** Urinary and intratumoral microbiome in patients with bladder cancer and normal controls.

Study	Sample	Urinary microbiome components(abundance and richness)	Note
Chipollini et al. 2020 (31)	Urine: 10 Normal 12 NMIBC 15 MIBC	Normal: enrichment of *Bacteroides*, *Lachnoclostridium*, and Burkholderiaceae family NMIBC: - MIBC: *Bacteroides* and *Faecalbacterium*	-
Mansour et al. 2020 (38)	Urine: 5 males 5 females Mucosal samples from BCa patients: 5 males 5 females	Urine: *Lactobacillus*, *Corynebacterium*, *Streptococcus*, *Staphylococcus* Tissue: *Bacteroides*, *Akkermansia*, *Klebsiella*, *Clostridium sensu stricto* Both: Firmicutes, Actinobacteria, Proteobacteria, Bacteroidetes, Cyanobacteria	The main bacterial phyla were largely conserved between sample types, but genera vary subtly between urine and tissue samples.
Sun et al. 2023 (30)	Tissue samples: 7 NMIBC 15 MIBC	NMIBC: *Ralstonia* (22.16%), *Cutibacterium* (6.6%), *Bacteroides* (5.51%), *Staphylococcus* (5.27%), *Acinetobacter* (5.07%)MIBC: *Ralstonia* (56.29%), *Cutibacterium* (9.82%), *Enterococcus* (6.91%), *Sphingomonas* (5.77%), *Metamycoplasma* (4.6%)	165 bacterial species in common between subtypes.265 species unique to NMIBC and 97 unique to MIBC.
Bukavina et al. 2023 (35)	Urine samples: 31 Normal 29 BCa tissues (validation cohort)	Normal: *Alloscardovia*, *Lactobacillus*, and the order Bifidobacteriales BCa: orders Clostridiales, Enterobacterales, and Bacteroidales	-
Sheng et al. 2025 (37)	Paired urine samples: 39 Primary BCa 39 Recurrent BCa	Recurrent BCa: enrichment of Firmicutes and loss of Bacteroidetes compared to primary BCa patients.	Recurrent BCa: urinary microenvironment dominated by specific microbial taxa
Li et al. 2025 (36)	Urine samples: 94 Normal: 41 males 53 females 64 BCa: 58 males 6 females	MIBC: predominance of *Peptoniphilus spp.* - associated with opportunistic urinary tract infections, chronic inflammation, and carcinogenesis	Bacterial enrichment: highest in men with BCa → women with BCa → healthy men → healthy women.

NMIBC: non-muscle-invasive bladder cancer; MIBC: muscle-invasive bladder cancer; BCa: bladder cancer; (-) no additional information.

## Microbiome-driven inflammatory mechanisms

Inflammation plays a complex role in cancer biology: while it can trigger an antitumor immune response, it may also become chronically established, thereby promoting carcinogenesis (39). In the bladder, the glycosaminoglycan (GAG) barrier acts as a physical separation among the urothelium, urine, and microorganisms (40). Bacterial virulence factors, including elastases, collagenases, and hyaluronidases, can degrade components of the extracellular matrix (41), facilitating microbial invasion of the tissue and the subsequent initiation and maintenance of inflammatory processes. Some bacterial virulence factors promote DNA damage, as exemplified by colibactin produced by *Escherichia coli* B2 and *Klebsiella pneumoniae*, which has been associated with chronic inflammation and genomic instability (42). Also, chronic inflammation involves interactions between the epithelium and bacterial biofilms, especially in the context of disruption of the GAG barrier (43).

Tumor necrosis factor-α (TNF-α) and interleukin-6 (IL-6) are key mediators of a pro-inflammatory tumor microenvironment. Increased levels of these cytokines are linked to poorer BCa prognosis, tumor progression via activation of the Janus kinase/signal transducer and activator of transcription 3 (JAK-STAT3) and nuclear factor kappa-light-chain-enhancer of activated B cells (NF-κB) signaling pathways, and tumor recurrence (44,45). Furthermore, interleukin-17 (IL-17) acts synergistically with IL-6 to further activate STAT3, stimulate angiogenesis, and facilitate tumor cell evasion to senescence (46). A co-culture study of *Eubacterium sp*. (common in non-invasive tumors) with bladder cancer organoids suggests that this bacterium may trigger the ECM1/ERK1/2/MMP9 phosphorylation pathway, enhancing cell proliferation and contributing to NMIBC progression (47).

In addition to these pathways, the inflammatory microenvironment is regulated by microbe-associated molecular patterns, which activate Toll-like receptors (TLRs) and subsequently the pro-tumoral signaling pathways, including NF-κB, PI3K-Akt-mTOR, and JAK-STAT3. The genera *Bacteroides* and *Enterococcus*, which have been detected in BCa tissues, were previously investigated in the gut, where they promoted the activation of β-catenin, IL-17R, NF-κB, and STAT3 pathways, facilitating malignant transformation (48). The Wnt/β-catenin pathway plays a central role in BCa progression (49).

The presence of certain commensal microbiome bacteria may reduce the incidence of urinary tract infections (UTIs) by modulating pro-inflammatory pathways, such as NF-κB, IL-6, and IL-8 (50). Microbiome components may also contribute to a pro-inflammatory microenvironment by metabolizing genotoxins (e.g., acetaldehyde and dietary nitrosamines), hormones, and bile acids (51). *Fusobacterium nucleatum* is an opportunistic pathogen identified in approximately 26% of BCa samples (28). In colorectal cancer, it has been well characterized for its ability to adhere to the epithelium via cell surface proteins such as FadA, Fap2, and RadD. This interaction activates the β-catenin pathway, induces pro-inflammatory cytokines and the NF-κB pathway, and promotes cell proliferation (52). Furthermore, *F. nucleatum* has been implicated in inhibiting natural killer (NK) and T cells, thereby enabling immune evasion (53).

External factors, such as smoking, introduce polycyclic aromatic hydrocarbons into urine. These compounds may serve as a carbon source for bacteria like *Enterococcus* and *Acinetobacter*, altering the microenvironment and promoting the proliferation of cancer-associated microorganisms (35). Also, *E. coli* infection has been associated with NF-κB pathway activation in BCa, supporting the development of an inflammatory microenvironment (22).

**Figure 1:**
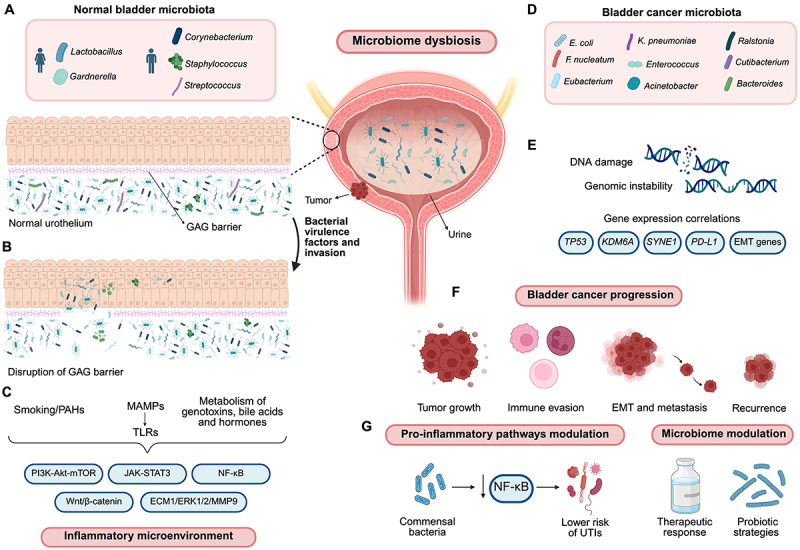
The role of the urinary microbiome in the pathogenesis, progression, and modulation of bladder cancer. Schematic overview of inflammatory microenvironment promoted by bladder cancer-associated microbiome, its role in pro- and anti-inflammatory pathways modulation, and therapeutic strategies. **A**, Under homeostatic conditions, the urinary microbiota is predominantly composed of *Lactobacillus* and *Gardnerella* in women and *Corynebacterium*, *Staphylococcus*, and *Streptococcus* in men, which is delimited from the bladder epithelium by a glycosaminoglycan (GAG) barrier. **B**, The production of virulence factors by pathogenic and/or opportunistic bacteria can disrupt this protective barrier, facilitating bacterial invasion, biofilm formation, DNA damage, and the initiation of inflammatory processes. **C**, Microorganism-associated molecular patterns (MAMPs), which activate Toll-like receptors (TLRs), together with the metabolism of genotoxins, hormones, bile acids, and external factors, such as tobacco-derived polycyclic aromatic hydrocarbons (PAHs), orchestrate the activation of pro-inflammatory and pro-tumorigenic signaling pathways, including NF-κB, PI3K-Akt-mTOR, JAK-STAT3, Wnt/β-catenin, and ECM1/ERK1/2/MMP9. Activation of these pathways establishes an inflammatory microenvironment. **D**, The bladder cancer-associated microbiome is mainly composed of *Escherichia coli*, *Fusobacterium nucleatum*, *Eubacterium*, *Klebsiella pneumoniae*, *Enterococcus*, *Acinetobacter*, *Ralstonia*, *Cutibacterium*, and *Bacteroides*. **E**, Dysbiosis of the microbiome and chronic bladder inflammation may promote DNA damage and genomic instability. These factors were correlated with a differential expression of several genes, including *TP53*, *KDM6A*, *SYNE1*, *PD-L1*, and genes associated with epithelial-mesenchymal transition (EMT). **F**, These dysbiotic bacterial taxa were previously associated with tumor development, immune evasion, epithelial-mesenchymal transition, tumor metastasis, and tumor recurrence. **G**, Conversely, commensal bacteria, such as Lactobacillus, help regulate pro-inflammatory pathways, including NF-κB, thereby decreasing the risk of urinary tract infections (UTIs). Moreover, bladder cancer and its urinary microbiome may be modulated by probiotic-based strategies, which were correlated with either improved or impaired therapeutic responses in bladder cancer patients. Created in BioRender. Cortes, J. (2026) <https://BioRender.com/nco5qum>.

Chronic or recurrent infections are well-established risk factors for BCa, as exemplified by human papillomavirus (HPV) infection (which induces inflammation, gene mutations, and DNA damage), herpes simplex virus (HSV) (which can interact with HPV), and the BK polyomavirus (54). *S. haematobium* infections stimulate the carcinogenic process through the establishment of persistent inflammation accompanied by bacteriuria in infected hosts (55). UTIs are commonly associated with poorer outcomes in patients with bladder cancer (56). However, a large study by Vermeulen and colleagues (57) found that a limited number of UTIs when treated with antibiotics may offer protection against BCa. The inflammatory process triggered by microbiome dysbiosis is illustrated in Figure 1.

## The microbiome and tumor progression

The microbiome also influences tumor progression by modulating the epithelial-mesenchymal transition (EMT), a process in which epithelial cells acquire an invasive phenotype (Figure 1). A strong association between the abundance of *E. coli* and *Saccharomonospora viridis* and the expression of EMT-linked genes was reported by Li et al. (58). The authors also observed that the expression of the elastin protein was positively associated with *Streptococcus gordonii* and negatively associated with *Burkholderia vietnamiensis*.

In 2024, Li and colleagues (59) performed an *in silico* analysis on 402 samples from The Cancer Genome Atlas (TCGA-BLCA), grouping BCa patients into three categories based on expression of EMT-associated genes (high, medium, and low). Although with distinct relative abundance, all groups presented Proteobacteria, Firmicutes, Actinobacteria, and Bacteroidetes. Proteobacteria showed a significant reduction in EMT-medium compared to the other groups. Bacteroidetes showed reduced abundance in EMT-high compared to EMT-medium. Linear discriminant analysis of effect size revealed 25 microbial signatures, with an emphasis on the high abundance of the genera *Terrabacter*, *Acinetobacter*, and *Lachnoclostridium* in EMT-high; *Lactobacillus*, *Bacillus*, and *Mycobacterium* in EMT-medium; and *Acidibacillus* and *Cyanothece* in EMT-low. By correlating these microbial signatures with EMT-related genes, five genera (*Terrabacter*, *Flammeovirga*, *Lachnoclostridium*, *Gallibacterium*, and *Sutterella*) were identified as abundant in EMT-high, showing a strong positive correlation with EMT genes. Among these, *Lachnoclostridium* and *Sutterella* are components of gut microbiota (60,61). *Lachnoclostridium* potentially contributes to increased expression of *C3AR1* (inflammatory response) and *EMP3* (EMT). *Sutterella* appears to positively regulate the expression of *CD14* and *COL6A2*. The EMT-high group exhibited the highest expression of immune checkpoint genes, immune infiltration, and possibly reduced sensitivity to immunotherapy, as well as a worse prognosis compared to the other groups. The abundance of *Lachnoclostridium* and *Sutterella* suggested that intestinal bacteria migrate to bladder tumor tissues (59).

These findings demonstrated the role of the microbiome in activating genes associated with invasion and tumor progression in bladder cancer.

## Bacillus Calmette-Guérin response and probiotic strategies for bladder cancer treatment

Patients with intermediate-to-high-risk NMIBC often have greater benefit from intravesical immunotherapy with Bacillus Calmette-Guérin (BCG) instillation. BCG is composed of an attenuated strain of *Mycobacterium bovis,* the gold standard treatment for preventing tumor recurrence since 1976 (62). In the clinical setting, BCG therapy for NMIBC stimulates the immune system and is effective in approximately 60% of patients (63). Among the alternatives for BCG non-responsive patients are therapeutics used for muscle-invasive disease.

The precise mechanism by which BCG interacts with the immune system is still unclear. Multiple immunological processes are apparently involved, including infection of urothelial cells by BCG via binding to fibronectin (an extracellular matrix protein), activation of the innate immune system, and, consequently, activation of immune cells (such as macrophages and helper T lymphocytes) and cytokine release. In addition, the adaptive system can be activated through the uptake of the bacterium by dendritic cells, which recognize the antigen and present it to CD4+ and CD8+ T lymphocytes. This cascade ultimately promotes the release of cytokines that induce apoptosis and necrosis of tumor cells (64).

The microbiome composition can affect how patients respond to BCG therapy (Figure 1). The predominance of *Actinomycetes* in the female microbiota, which resembles the composition of BCG, may help explain the lower incidence of BCa in women (65). Moreover, commensal bacteria such as *Lactobacillus iners* can bind to BCG sites (e.g., fibronectin and α5β1 integrins), potentially promoting saturation and reducing the effectiveness of BCG in some BCa patients (66). These findings suggest that *Lactobacillus* could be a potential target for BCa treatment and an alternative to BCG.

Longitudinal studies have indicated that the urinary microbiome can be reconfigured into a pro-inflammatory state in certain patients, which is associated with immunological tolerance and/or exhaustion, and paradoxically with lower survival and a higher risk of tumor progression. Hussein et al. (67) verified a higher proportion of the genera *Serratia*, *Pseudomonas*, *Brochothrix*, and *Negativicoccus* in BCG responders with NMIBC. Conversely, Sweis et al. (68) reported an enrichment of Proteobacteria, particularly *Gammaproteobacteria* in NMIBC patients who experienced recurrence, whereas patients without recurrence showed a higher abundance of Firmicutes, such as the Lactobacillales order.

A recent multicenter Brazilian study analyzed urine samples from 32 male patients with intermediate-to-high risk NMIBC and 41 with benign prostatic hyperplasia as controls (69). The authors found that BCG treatment did not significantly change the diversity or composition of the bladder microbiota. However, BCG was more effective when higher levels of *Lactobacillus*, *Streptococcus*, and *Cutibacterium* were present before treatment. No significant differences were found between the control and NMIBC groups. Other studies have also reported that greater microbial diversity before BCG instillation is associated with a better therapeutic response and enhanced immune activation (70).

Modulating the microbiome is an efficient strategy for BCa control (Figure 1). Probiotics and their metabolites have been extensively studied for the treatment of inflammation, diarrhea, hypercholesterolemia, urogenital infections, and obesity (71). Their mechanisms include modulation of the innate and adaptive immune systems to control inflammation (e.g., in colitis); regulation of gene expression; and interactions of their metabolites with carcinogens (72). As a result, the antitumor effects include the induction of apoptosis through the stimulation of pro-apoptotic protein production (e.g., Bcl-2-associated X protein) and caspase activation, the reduction of anti-apoptotic proteins, activation of tumor suppressor genes, prevention of metastasis, and the regulation of cellular signaling pathways (72).

The genera *Lactobacillus* presents the greatest probiotic versatility. *Lactobacillus rhamnosus* GG and *Lactobacillus casei* Shirota have demonstrated antitumor effects in bladder cancer cells by inhibiting proliferation, inducing cytotoxicity, and promoting necrosis (73). The intravesical instillation of *Lactobacillus casei* Shirota proved to be a protective method for BCa incidence, yielding more promising results than BCG instillation in a murine BCa model (74).

The supplementation of *Butyricicoccus pullicaecorum*, a butyrate-producing bacterium, has demonstrated an antitumor role in urothelial cells (75). This bacterium acts by inducing the increased expression of short-chain fatty acid receptors (*GPR109B* and *GPR43*) and the *FABP4* transporter, contributing to cell cycle regulation, growth control, apoptosis, and gene expression in urothelial cells (75). Similarly, intravesical administration of *Clostridium butyricum* promoted tumor cell growth by inducing apoptosis via release of tumor necrosis factor-related apoptosis-inducing ligand (72). Also, the regular consumption of lactic acid bacteria, such as those in fermented dairy products, has been associated with a protective role against BCa incidence (72).

## Transcriptome and metagenomic correlation: a new perspective to bladder cancer prognosis and treatment

Zhang et al. (34) developed a prognostic signature for BCa based on eleven core genes identified from the overlap of immunity and antimicrobial-related genes and differentially expressed genes from TCGA-BLCA, with a focus on the immune checkpoint PD-L1. The model stratifies patients into high- and low-risk groups, with the high-risk group exhibiting a significantly worse prognosis. Analysis of the tumor microenvironment showed distinct microbial and immunological profiles between the groups. The low-risk group had higher levels of *Sanguibacter*, *Elizabethkingia*, *Actinobacillus*, and *Spirochaeta*. The phylum Aquificae was more prevalent in the high-risk group, which also demonstrated lower infiltration of CD8^+^ T cells and macrophages. Beneficial microorganisms such as *Butyrivibrio* and *Arthrospira* predominated in the low-risk group, whereas the high-risk group included less-studied genera, including *Oleomonas*, *Cellulomonas*, *Candidatus Paracaedibacter*, *Prosthecobacter*, *Aphanizomenon*, *Thiothrix*, and *Cycloclasticus* (34).

A microbial scoring system (MS) was developed using six microorganisms: *Syntrophobotulus*, *Granulicatella*, *Xanthomonas*, *Pseudoalteromonas*, the phyla Aquificae, and *Niabella*. A higher abundance of the first four was associated with a worse prognosis, whereas *Niabella* was linked to improved survival (34). Aquificae were more abundant in stage I, suggesting a potential association between the intratumoral microbiota and clinical stage. The MS-low group showed a better prognosis. CIBERSORT analysis was used to characterize cell subsets, revealing an enrichment of memory B cells in the MS-high group, and a higher tumor mutational burden in the MS-low group (90.68 *vs* 85.06%). The MS-low group showed higher frequencies of *TP53* and *KDM6A* mutations, while *SYNE1* mutations were more prevalent in the MS-high group. The MS-low group also showed increased expression of immune checkpoints (e.g., *BTN2A2*, *BTN3A1*, *CD96*, *PD-L1*, *CEACAM1*, *HLA-C*, and *HLA-G*). The authors observed greater microbial diversity in bladder cancer samples compared to normal bladder tissues, underscoring the need for further research on the role of the tumor-associated microbiota in bladder cancer development, prognosis, and treatment (34).

Chen et al. (76) examined the relationship between urogenital microbiota and PD-L1 expression in male patients, analyzing 28 NMIBC samples and 31 urine samples (11 PD-L1 positive, 20 negative). Samples were grouped by PD-L1 status. The PD-L1-positive group showed greater bacterial richness, which has been identified as a potential marker for prognosis and tumor progression (76). Microbiota composition differed by PD-L1 expression, with *Leptotrichia* more abundant in the positive group and *Prevotella* in the negative group. *Leptotrichia* has been linked to various cancers and was associated with pro-inflammatory cytokines such as IL-6 and IFN-γ, which may promote PD-L1 expression and support tumor immune evasion (77,78). In contrast, *Roseomonas* and *Propionibacterium,* which were also enriched in the positive group, have been reported to confer benefits. *Roseomonas* mucosa supports immune balance (79), while the *Propionibacterium* strain P. UF1 increases colonic Th17 and Treg cells, offering protection against pro-inflammatory diseases (80).

In the study by Gilbert et al. (81), exposure to *Gardnerella*, one of the most abundant members of the female urinary microbiota, was associated with altered expression of genes linked to immune activation. RNA sequencing analyses revealed that repeated exposures activated genes and pathways linked to DNA damage, programmed cell death, urothelial differentiation and proliferation, and immune and inflammatory responses. The authors identified 38 genes with increased expression and 11 with reduced expression after exposure to the pathogen, with the enrichment of pathways associated with inflammation, immunity, and urothelial turnover. Distinctly, the genes *CXCL5*, *CXCR2*, *PTX3*, *CLEC4E,* and *TREM1* were implicated in promoting inflammatory responses. Furthermore, genes related to EMT (such as *CXCL5*, *CXCR2*, and *TFF1*) showed increased expression, while *ANXA10*, *FOSL1*, *KRT6A,* and *MMP10* were associated with bladder cancer development. These findings corroborate the role of urinary microbiome components in promoting biological effects that alter bladder gene expression (81) (Figure 1).

## Future directions

Substantial progress has been made in recent years regarding the composition of the urinary microbiome and the impact of microbial dysbiosis in the development, progression, recurrence, and therapeutic response of bladder cancer. However, studies analyzing both BCa tissue and paired tumor-urine samples remain limited, which restricts direct comparisons of microbial composition across biological compartments. While some studies support the use of probiotics for the treatment or prevention of BCa and other cancers, clinical adoption remains limited, underscoring the need for larger, multicenter trials.

Organoid models offer a promising platform for personalized therapy by enabling microbiome analysis, patient-specific comparisons, and therapeutic testing, including probiotics. Overall, microbiome profiling is a highly valuable tool in bladder cancer research. Further investigations into tumor- and urine-associated microbiota are needed to improve treatment and surveillance of disease recurrence.

## Data Availability

The entire dataset supporting the results of this study was published in the article itself.

## References

[1] Bray F, Laversanne M, Sung H, Ferlay J, Siegel RL, Soerjomataram I (2024). Global cancer statistics 2022: GLOBOCAN estimates of incidence and mortality worldwide for 36 cancers in 185 countries. CA Cancer J Clin.

[2] Çivi Çetin K, Öner S, Erdoğan B, Simşek G, Sezer M (2023). Young population bladder neoplasms. Eur Rev Med Pharmacol Sci.

[3] Pignot G, Barthélémy P, Borchiellini D (2024). Sex disparities in bladder cancer diagnosis and treatment. Cancers (Basel).

[4] Doshi B, Athans SR, Woloszynska A (2023). Biological differences underlying sex and gender disparities in bladder cancer: current synopsis and future directions. Oncogenesis.

[5] Freedman ND, Silverman DT, Hollenbeck AR, Schatzkin A, Abnet CC (2011). Association between smoking and risk of bladder cancer among men and women. JAMA.

[6] Bellamri M, Walmsley SJ, Brown C, Brandt K, Konorev D, Day A (2022). DNA damage and oxidative stress of tobacco smoke condensate in human bladder epithelial cells. Chem Res Toxicol.

[7] Gabriel U, Li L, Bolenz C, Steidler A, Kränzlin B, Saile M (2012). New insights into the influence of cigarette smoking on urothelial carcinogenesis: smoking-induced gene expression in tumor-free urothelium might discriminate muscle-invasive from nonmuscle-invasive urothelial bladder cancer. Mol Carcinog.

[8] Cumberbatch MGK, Cox A, Teare D, Catto JWF (2015). Contemporary occupational carcinogen exposure and bladder cancer: a systematic review and meta-analysis. JAMA Oncol.

[9] Chaudhary P, Singha B, Abdel-Hafiz HA, Velegraki M, Sundi D, Satturwar S (2025). Sex differences in bladder cancer: understanding biological and clinical implications. Biol Sex Differ.

[10] Dancik GM, Owens CR, Iczkowski KA, Theodorescu D (2014). A cell of origin gene signature indicates human bladder cancer has distinct cellular progenitors. Stem Cells.

[11] Netto GJ, Amin MB, Berney DM, Compérat EM, Gill AJ, Hartmann A (2022). The 2022 World Health Organization classification of tumors of the urinary system and male genital organs-Part B: prostate and urinary tract tumors. Eur Urol.

[12] Willis D, Kamat AM (2015). Nonurothelial bladder cancer and rare variant histologies. Hematol Oncol Clin North Am.

[13] Kamat AM, Hahn NM, Efstathiou JA, Lerner SP, Malmström PU, Choi W (2016). Bladder cancer. Lancet.

[14] Gontero P, Birtle A, Capoun O, Compérat E, Dominguez-Escrig JL, Liedberg F (2024). European Association Of Urology Guidelines on non-muscle-invasive bladder cancer (TaT1 and carcinoma *in situ*)-a summary of the 2024 guidelines update. Eur Urol.

[15] Holzbeierlein JM, Bixler BR, Buckley DI, Chang SS, Holmes R, James AC (2024). Diagnosis and treatment of non-muscle invasive bladder cancer: AUA/SUO guideline: 2024 amendment. J Urol.

[16] Mitra AP, Cai J, Miranda G, Bhanvadia S, Quinn DI, Schuckman AK (2022). Management trends and outcomes of patients undergoing radical cystectomy for urothelial carcinoma of the bladder: evolution of the University of Southern California experience over 3,347 cases. J Urol.

[17] Claps F, Biasatti A, Di Gianfrancesco L, Ongaro L, Giannarini G, Pavan N (2024). The prognostic significance of histological subtypes in patients with muscle-invasive bladder cancer: an overview of the current literature. J Clin Med.

[18] Dyrskjøt L, Hansel DE, Efstathiou JA, Knowles MA, Galsky MD, Teoh J (2023). Bladder cancer. Nat Rev Dis Primer.

[19] Hurst CD, Cheng G, Platt FM, Castro MAA, Marzouka NADS, Eriksson P (2021). Stage-stratified molecular profiling of non-muscle-invasive bladder cancer enhances biological, clinical, and therapeutic insight. Cell Rep Med.

[20] Robertson AG, Kim J, Al-Ahmadie H, Bellmunt J, Guo G, Cherniack AD (2017). Comprehensive molecular characterization of muscle invasive bladder cancer. Cell.

[21] Spooner R, Yilmaz Ö (2011). The role of reactive-oxygen-species in microbial persistence and inflammation. Int J Mol Sci.

[B22] El-Mosalamy H, Salman TM, Ashmawey AM, Osama N (2012). Role of chronic E. coli infection in the process of bladder cancer- an experimental study. Infect Agent Cancer.

[23] Wong-Rolle A, Wei HK, Zhao C, Jin C (2021). Unexpected guests in the tumor microenvironment: microbiome in cancer. Protein Cell.

[24] Lewis DA, Brown R, Williams J, White P, Jacobson SK, Marchesi JR (2013). The human urinary microbiome; bacterial DNA in voided urine of asymptomatic adults. Front Cell Infect Microbiol.

[25] Pearce MM, Zilliox MJ, Rosenfeld AB, Thomas-White KJ, Richter HE, Nager CW (2015). The female urinary microbiome in urgency urinary incontinence. Am J Obstet Gynecol.

[26] Shrestha E, White JR, Yu SH, Kulac I, Ertunc O, De Marzo AM (2018). Profiling the urinary microbiome in men with positive versus negative biopsies for prostate cancer. J Urol.

[27] Markowski MC, Boorjian SA, Burton JP, Hahn NM, Ingersoll MA, Maleki Vareki S (2019). The microbiome and genitourinary cancer: a collaborative review. Eur Urol.

[28] Bučević Popović V, Šitum M, Chow CET, Chan LS, Roje B, Terzić J (2018). The urinary microbiome associated with bladder cancer. Sci Rep.

[29] Pederzoli F, Ferrarese R, Amato V, Locatelli I, Alchera E, Lucianò R (2020). Sex-specific alterations in the urinary and tissue microbiome in therapy-naïve urothelial bladder cancer patients. Eur Urol Oncol.

[30] Sun JX, Xia QD, Zhong XY, Liu Z, Wang SG (2023). The bladder microbiome of NMIBC and MIBC patients revealed by 2bRAD-M. Front Cell Infect Microbiol.

[31] Chipollini J, Wright JR, Nwanosike H, Kepler CY, Batai K, Lee BR (2020). Characterization of urinary microbiome in patients with bladder cancer: Results from a single-institution, feasibility study. Urol Oncol.

[B32] Liu F, Liu A, Lu X, Zhang Z, Xue Y, Xu J (2019). Dysbiosis signatures of the microbial profile in tissue from bladder cancer. Cancer Med.

[B33] Wu P, Zhang G, Zhao J, Chen J, Chen Y, Huang W (2018). Profiling the urinary microbiota in male patients with bladder cancer in China. Front Cell Infect Microbiol.

[34] Zhang Y, Lin H, Liang L, Jin S, Lv J, Zhou Y (2024). Intratumoral microbiota as a novel prognostic indicator in bladder cancer. Sci Rep.

[35] Bukavina L, Isali I, Ginwala R, Sindhani M, Calaway A, Magee D (2023). Global meta-analysis of urine microbiome: colonization of polycyclic aromatic hydrocarbon-degrading bacteria among bladder cancer patients. Eur Urol Oncol.

[B36] Li N, Wang L, Yang Q, Li F, Shi Z, Feng X (2025). Identification and evaluation of the urinary microbiota associated with bladder cancer. Cancer Innov.

[B37] Sheng Z, Xu J, Wang M, Xu X, Zhu J, Zeng S (2025). The role of urinary microbiota in primary and recurrent bladder cancer: insights from a propensity score matching study. BMC Cancer.

[38] Mansour B, Monyók Á, Makra N, Gajdács M, Vadnay I, Ligeti B (2020). Bladder cancer-related microbiota: examining differences in urine and tissue samples. Sci Rep.

[39] Hanahan D, Weinberg RA (2011). Hallmarks of cancer: the next generation. Cell.

[40] Parsons CL, Boychuk D, Jones S, Hurst R, Callahan H (1990). Bladder surface glycosaminoglycans: an epithelial permeability barrier. J Urol.

[41] Alfano M, Canducci F, Nebuloni M, Clementi M, Montorsi F, Salonia A (2016). The interplay of extracellular matrix and microbiome in urothelial bladder cancer. Nat Rev Urol.

[42] Bilski K, Dobruch J, Kozikowski M, Skrzypczyk MA, Oszczudłowski M, Ostrowski J (2020). Urobiome in gender-related diversities of bladder cancer. Int J Mol Sci.

[43] Nadler N, Kvich L, Bjarnsholt T, Jensen JB, Gögenur I, Azawi N (2021). The discovery of bacterial biofilm in patients with muscle invasive bladder cancer. APMIS.

[44] Wigner P, Grębowski R, Bijak M, Saluk-Bijak J, Szemraj J (2021). The interplay between oxidative stress, inflammation and angiogenesis in bladder cancer development. Int J Mol Sci.

[45] Kumari N, Agrawal U, Mishra AK, Kumar A, Vasudeva P, Mohanty NK (2017). Predictive role of serum and urinary cytokines in invasion and recurrence of bladder cancer. Tumour Biol.

[46] Wang L, Yi T, Kortylewski M, Pardoll DM, Zeng D, Yu H (2009). IL-17 can promote tumor growth through an IL-6-Stat3 signaling pathway. J Exp Med.

[47] Zhang Y, Wang W, Zhou H, Cui Y (2023). Urinary *Eubacterium sp*. CAG:581 promotes non-muscle invasive bladder cancer (NMIBC) development through the ECM1/MMP9 pathway. Cancers.

[48] Peng Z, Zhuang J, Shen B (2023). The role of microbiota in tumorigenesis, progression and treatment of bladder cancer. Microbiome Res Rep.

[49] Kotolloshi R, Gajda M, Grimm MO, Steinbach D (2022). Wnt/β-catenin signalling and its cofactor BCL9L have an oncogenic effect in bladder cancer cells. Int J Mol Sci.

[50] Cosseau C, Devine DA, Dullaghan E, Gardy JL, Chikatamarla A, Gellatly S (2008). The commensal *Streptococcus salivarius* K12 downregulates the innate immune responses of human epithelial cells and promotes host-microbe homeostasis. Infect Immun.

[51] Mantovani A, Allavena P, Sica A, Balkwill F (2008). Cancer-related inflammation. Nature.

[52] Wu J, Li Q, Fu X (2019). *Fusobacterium nucleatum* contributes to the carcinogenesis of colorectal cancer by inducing inflammation and suppressing host immunity. Transl Oncol.

[53] Gur C, Ibrahim Y, Isaacson B, Yamin R, Abed J, Gamliel M (2015). Binding of the Fap2 protein of Fusobacterium nucleatum to human inhibitory receptor TIGIT protects tumors from immune cell attack. Immunity.

[54] Yao X, Xu Z, Duan C, Zhang Y, Wu X, Wu H (2023). Role of human papillomavirus and associated viruses in bladder cancer: an updated review. J Med Virol.

[55] Ashour DS, Othman AA (2020). Parasite-bacteria interrelationship. Parasitol Res.

[56] Richards KA, Ham S, Cohn JA, Steinberg GD (2016). Urinary tract infection-like symptom is associated with worse bladder cancer outcomes in the Medicare population: Implications for sex disparities. Int J Urol.

[57] Vermeulen SH, Hanum N, Grotenhuis AJ, Castaão-Vinyals G, van der Heijden AG, Aben KK (2015). Recurrent urinary tract infection and risk of bladder cancer in the Nijmegen bladder cancer study. Br J Cancer.

[58] Li WT, Iyangar AS, Reddy R, Chakladar J, Bhargava V, Sakamoto K (2021). The bladder microbiome is associated with epithelial-mesenchymal transition in muscle invasive urothelial bladder carcinoma. Cancers (Basel).

[59] Li Q, Sun Y, Zhai K, Geng B, Dong Z, Ji L (2024). Microbiota-induced inflammatory responses in bladder tumors promote epithelial-mesenchymal transition and enhanced immune infiltration. Physiol Genomics.

[60] Zhang W, Zou G, Li B, Du X, Sun Z, Sun Y (2020). Fecal microbiota transplantation (FMT) alleviates experimental colitis in mice by gut microbiota regulation. J Microbiol Biotechnol.

[61] Paramsothy S, Nielsen S, Kamm MA, Deshpande NP, Faith JJ, Clemente JC (2019). Specific bacteria and metabolites associated with response to fecal microbiota transplantation in patients with ulcerative colitis. Gastroenterology.

[62] Morales A, Eidinger D, Bruce AW (1976). Intracavitary Bacillus Calmette-Guerin in the treatment of superficial bladder tumors. J Urol.

[63] Zlotta AR, Fleshner NE, Jewett MA (2009). The management of BCG failure in non-muscle-invasive bladder cancer: an update. Can Urol Assoc J.

[64] Lidagoster S, Ben-David R, De Leon B, Sfakianos JP (2024). BCG and alternative therapies to BCG therapy for non-muscle-invasive bladder cancer. Curr Oncol.

[65] Raoult D (2017). Is there a link between urinary microbiota and bladder cancer?. Eur J Epidemiol.

[66] McMillan A, Macklaim JM, Burton JP, Reid G (2013). Adhesion of *Lactobacillus iners* AB-1 to human fibronectin: a key mediator for persistence in the vagina?. Reprod Sci.

[67] Hussein AA, Elsayed AS, Durrani M, Jing Z, Iqbal U, Gomez EC (2021). Investigating the association between the urinary microbiome and bladder cancer: an exploratory study. Urol Oncol.

[68] Sweis RF, Golan S, Barashi N, Hill E, Andolfi C, Werntz RP (2019). Association of the commensal urinary microbiome with response to Bacillus Calmette-Guérin (BCG) immunotherapy in nonmuscle invasive bladder cancer. J Clin Oncol.

[69] Heidrich V, Mariotti ACH, Inoue LT, Coser EM, Dos Santos EX, Dos Santos HDB (2024). The bladder microbiota is not significantly altered by intravesical BCG therapy. Urol Oncol.

[70] Bourgi A, Bruyàre F, Rusch E (2025). Microbiome and immunotherapy in bladder cancer: the missing link. Fr J Urol.

[71] Sankarapandian V, Venmathi Maran BA, Rajendran RL, Jogalekar MP, Gurunagarajan S, Krishnamoorthy R (2022). An update on the effectiveness of probiotics in the prevention and treatment of cancer. Life (Basel).

[72] Dadgar-Zankbar L, Mokhtaryan M, Bafandeh E, Javanmard Z, Asadollahi P, Darbandi T (2025). Microbiome and bladder cancer: the role of probiotics in treatment. Future Microbiol.

[73] Seow SW, Rahmat JNB, Mohamed AAK, Mahendran R, Lee YK, Bay BH (2002). *Lactobacillus* species is more cytotoxic to human bladder cancer cells than *Mycobacterium bovis* (bacillus Calmette-Guerin). J Urol.

[74] Takahashi T, Kushiro A, Nomoto K, Uchida K, Morotomi M, Yokokura T (2001). Antitumor effects of the intravesical instillation of heat killed cells of the *Lactobacillus casei* strain Shirota on the murine orthotopic bladder tumor MBT-2. J Urol.

[75] Wang YC, Ku WC, Liu CY, Cheng YC, Chien CC, Chang KW (2021). Supplementation of probiotic *Butyricicoccus pullicaecorum* mediates anticancer effect on bladder urothelial cells by regulating butyrate-responsive molecular signatures. Diagnostics (Basel).

[76] Chen C, Huang Z, Huang P, Li K, Zeng J, Wen Y (2022). Urogenital microbiota: potentially important determinant of PD-L1 expression in male patients with non-muscle invasive bladder cancer. BMC Microbiol.

[77] Jang JY, Song IS, Baek KJ, Choi Y, Ji S (2017). Immunologic characteristics of human gingival fibroblasts in response to oral bacteria. J Periodontal Res.

[78] Cha JH, Chan LC, Li CW, Hsu JL, Hung MC (2019). Mechanisms controlling PD-L1 expression in cancer. Mol Cell.

[79] Myles IA, Earland NJ, Anderson ED, Moore IN, Kieh MD, Williams KW (2018). First-in-human topical microbiome transplantation with *Roseomonas mucosa* for atopic dermatitis. JCI Insight.

[80] Colliou N, Ge Y, Sahay B, Gong M, Zadeh M, Owen JL (2017). Commensal *Propionibacterium* strain UF1 mitigates intestinal inflammation via Th17 cell regulation. J Clin Invest.

[81] Gilbert NM, O'Brien VP, Waller C, Batourina E, Mendelsohn CL, Lewis AL (2022). *Gardnerella* exposures alter bladder gene expression and augment uropathogenic *Escherichia coli* urinary tract infection in mice. Front Cell Infect Microbiol.

